# Injuries and Diseases of the Genital and Urinary Organs

**Published:** 1896-08

**Authors:** 


### Book Reviews.

Injuries and Diseases of the Genital and Urinary Organs. By Henry Morris, M. A., M. B., Lond., F. R. C. S., Surgeon to, and Lecturer on, Surgery at the Middlesex Hospital; Member of the Council and of the Court of Examiners of the Royal College of Surgeons, England, etc. Small 8vo, pp. xvi.—478 With ninety-seven illustrations. New York: William Wood & Co. 1895.

Dr. Morris adopted a systematic course in presenting the subject matter of his book, and describes "in a methodical manner all that is requisite to be known about this subject, basing his teaching not on his own experiences only, but also on that of others," thus following out the method maintained in his Surgical Diseases of the Kidneys. Clearness and completeness of description are not sacrificed to brevity; fundamentals are given while details are to be sought in more extensive works. The phenomena of urinary fever he considers to be more closely connected with the affections of the kidneys than with the lower urinary tract, and refers the reader to his Surgical Diseases of the Kidneys.

Part I. contains fourteen chapters on the Genital Organs. The fourteenth chapter is devoted to diseases and injuries of the vesiculæ seminales, in which he quotes Lloyd, who believes that inflammation of these organs is probably a more common result of gonnorrhea than epididymitis; also Velpeau, who expressed the opinion that inflammation of the seminal vesicles often arose from gonorrhea. Morris says it is generally overlooked and sometimes mistaken for prostatitis. He is evidently not familiar with the excellent work done by Fuller (New York). Nothing is said about stripping the vesicles.

Part II. treats of the genito-urinary organs which are concerned in both the functions of generation and urination—namely, the penis, urethra and prostate. The prostate is regarded as an organ connected with both of these functions, because (1) the common ejaculatory ducts pass through it; (2) it secretes a fluid which is added to and dilutes the semen at the time of ejaculation; (3) it surrounds the first part of the urethra; (4) it acts like a collar at the neck of the bladder. The part played by the prostate in the function of urination is not clearly stated. Circumcision is not regarded as an insignificant operation; care and judgment are required to prevent infection and to secure a good result.

Under the heading Folliculitis he states that hypersecretion of Littré's glands often follows urethritis. These aceni afford nests for gonococci, in which chronic discharge is maintained and where materials are generated which lead to recrudesence of the disease. Diday describes the urethra as a sieve in the holes of which gonorrhea extends and into which the remedies must penetrate. Tyson's glands in the coronal furrow may become affected.

Fabry reports a case in which he found a tumor the size of a lentil situated between the folds of the prepuce, from which he pressed a drop or two of liquid containing gonococci, yet this subject had not had gonorrhea for fifteen years. Gonorrhea persists in the glands long after the anterior urethra is dry, and this is especially true of the prostate and other glands of the deep urethra. Hence, bicycle and horseback riding, venery and various excesses frequently arouse this latent condition into pernicious activity.

Chapter eight gives a full exposé of the subject of stricture. Chapter seventeen treats of enlarged prostate. The various surgical procedures for the relief of senile hypertrophy, except ligation of the vasa deferentia, are clearly described. The views and results of castration for the relief of this malady, of several different authorities are given and he leaves the subject *sub judice*.

Part III. concerns the urinary bladder. Morris states that cystitis is extremely frequent as a result of a deep-seated urethritis, aggravated by mechanical violence or excesses. He refers to the fact that the mucous membrane of the bladder is very tolerant of microbic forms as apparent contradiction to the claim that microbic infections cause cystitis. It has been demonstrated that virulent microbes pass through the bladder without exciting cystitis, unless there be urinary stasis or lesion of the membrane. He affirms that forced injection or the passage of a catheter frequently provoke cystitis, and quotes the statement of Guyen that cystitis is characterised by three constant symptoms: frequent and painful micturition and pyuria; and that there is no elevation of temperature in uncomplicated cystitis.

In cases of acute and chronic gonorrhea he warns us of the danger of causing cystitis by forced injections and catheters.

Irrigation of the bladder is to be avoided in acute cystitis.

The reviewer differs from the learned author. His experience has taught him that forceless and catheterless irrigation of the acutely inflamed bladder is the treatment *par excellence.*

Diseases and Injuries of the Genital and Urinary Organs is illustrated with 100 cuts, some of which are original, and many authorities are quoted and credited. This volume is a very valuable addition to medical literature. B. H. D.

